# Microstructure Formation in Cast TiZrHfCoNiCu and CoNiCuAlGaIn High Entropy Shape Memory Alloys: A Comparison

**DOI:** 10.3390/ma12244227

**Published:** 2019-12-16

**Authors:** Tetiana A. Kosorukova, Gregory Gerstein, Valerii V. Odnosum, Yuri N. Koval, Hans Jürgen Maier, Georgiy S. Firstov

**Affiliations:** 1 G.V. Kurdyumov Institute for Metal Physics of the National Academy of Sciences of Ukraine, 36 Vernadsky blvd., 03680 Kiev, Ukraine; tatiana.kosorukova@gmail.com (T.A.K.); odnosum@imp.kiev.ua (V.V.O.); koval@imp.kiev.ua (Y.N.K.); 2 Institut für Werkstoffkunde (Materials Science) Leibniz Universität Hannover, An der Universität 2, 30823 Garbsen, Germany; gerstein@iw.uni-hannover.de (G.G.); maier@iw.uni-hannover.de (H.J.M.)

**Keywords:** dendritic liquation, martensitic transformation, high entropy shape memory alloys

## Abstract

The present study is dedicated to the microstructure characterization of the as-cast high entropy intermetallics that undergo a martensitic transformation, which is associated with the shape memory effect. It is shown that the TiZrHfCoNiCu system exhibits strong dendritic liquation, which leads to the formation of martensite crystals inside the dendrites. In contrast, in the CoNiCuAlGaIn system the dendritic liquation allows the martensite crystals to form only in interdendritic regions. This phenomenon together with the peculiarities of chemical inhomogeneities formed upon crystallization of this novel multicomponent shape memory alloys systems will be analyzed and discussed.

## 1. Introduction

The development of the so-called high entropy alloys (HEA) has triggered a substantial amount of materials research. It all started in 2004 with the concept explained in a publication by J.W. Yeh et al. [1], which has triggered a real breakthrough in the development of structural materials. This concept involves the creation of the multi-element metallic materials close to an equimolar composition, i.e., without a principal element. The entropy of mixing is higher compared to conventional materials and ensures a high phase stability [2,3,4,5,6]. At the same time, the development of the shape memory alloys experienced certain difficulties associated with functional degradation, especially when their envisaged application targeted elevated temperatures [7,8,9,10,11]. HEA are known for their non-obvious solution hardening [12,13]. Thus, it has been suggested that the application of the high entropy concept to shape memory alloys might help to overcome the difficulties mentioned above through the suppression of irreversible fatigue effects. The first successful attempt was made using the TiZrHfCuNiCo system. The design employed the B2 TiNi intermetallic compound as a prototype. Exclusion of Co from the equiatomic composition resulted in a martensitic transformation accompanied by perfect shape memory effect at elevated temperatures due to the significant two-fold increase in strength compared with binary TiNi [14]. It was shown that HEA properties were those that high temperature shape memory alloys needed for their improvement, and a number of six component high entropy shape memory alloys (HESMA) from the TiZrHfCoNiCu system were developed with enhanced yield strength of 1200 MPa and reversible strains up to 2% in the as-cast state [15]. The peculiar high temperature crystal structure—a triclinically distorted one of the B2 type that carries these distortions into the B19’ martensite phase (modeled with the help of ab-initio calculations and confirmed by X-ray diffraction measurements) [16]—was proposed to be the origin of the HESMA yield strength and shape memory behavior enhancement. In this context, it was shown that for the TiZrHfCoNiCu system a B2↔B19’ type of martensite transformation takes place [14,15,16]. Recently, an extra six component system CoNiCuAlGaIn, which emulates the B2 NiAl intermetallic compound was introduced [17]. In the as-cast state, it shows a B2 high temperature structure distorted similarly to TiZrHfCoNiCu and it also carries triclinic distortions into the tetragonal martensitic phase. TEM and DSC results clearly confirm the B2↔L1_0_ type of martensitic transformation [17]. Thus, the formation of multi-component intermetallic compounds with a distorted B2 structure undergoing a martensitic transformation appears as a physical regularity, which is important for subsequent shape memory behavior. It should be noted that very recently Lee et al. [18] managed to produce a solid solution CrMnFeCoNi HESMA that exhibit recovery strains of almost 2%. However, the phase transformation is accompanied by irreversible plastic strains of 1%–5.8%. In other words, shape recovery is incomplete, and with an increase of pre-strain from 0.9% to 7.6%, the shape recovery ratio drops from 89% to 24%. In fact, at the moment the only HESMA that exhibit complete shape recovery up to 2% are multi-component intermetallic compounds. One of the remarkable features of these HESMA is that they undergo a martensitic transformation and exhibit shape memory behavior in the as-cast state although this is characterized by strong dendritic liquation. Such chemical inhomogeneity is well known for cast HEA (e.g., [1] (p. 300), [2] (pp. 24–25), [3] (pp. 78–112), [13] (pp. 287–288), [19,20]), but was mainly observed and described for solid solutions. Thus, the present study was dedicated to the description and analysis of the microstructural peculiarities that appear after casting of intermetallic HESMA and how martensite formation is taking place under such conditions.

## 2. Materials and Methods

The Ti–Zr–Hf–Co–Ni–Cu and Co–Ni–Cu–Al–Ga–In alloys used in the present investigation were arc-melted from iodide Ti, Zr, and Hf, electrolytic Co, Ni, and Cu and Al, Ga, and In of high purity in a pre-gettered argon. Ingots were turned and re-melted nine times to ensure adequate homogeneity. The weight the ingots was typically 5–10 g. Scanning electron microscopy studies were carried out using a Zeiss SUPRA 55 VP field emitter scanning electron microscope (FE-SEM) with a lateral resolution 1.2 nm. For element analysis an EDX system Quantax (silicon drift detector SDD, Series 5010, Type 1108, 30 mm^2^, Collimator Zr on Chip, Aperture 3.5mm) from Bruker with an energy resolution of < 125 eV FWHM at MnK_α_ (Peakshift 5–300 kcps < 5 eV, at 60 kcps shaper, throughput 1.0 µs shaping time, 100 kcps input count rate) was employed. Smoothening of the elemental line scan data obtained in the SEM experiments on Co–Ni–Cu–Al–Ga–In alloy was performed using an FFT filter method (9 points window) implemented in the OriginPro 2015 software package. Differential thermal analysis (DTA) was performed on a Setaram LabSys 1600 system, calibrated using the phase transitions and melting temperatures of different pure metals. The measurements were performed at heating/cooling rates of 40 K min^−1^, under a 5N purity argon flow. The DTA baseline was subtracted using the PeakFit v. 4.12 software. The accuracy of the measurements for the temperatures of the phase transformations was ± 7K.

## 3. Results

**Figure 1:**
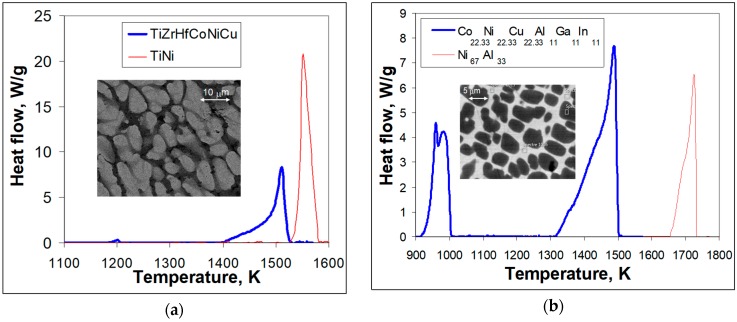
Heat flow upon crystallization from the liquid (40 K/min cooling rate) and the resulting cast microstructures: (**a**) red line—equiatomic TiNi and blue one—equiatomic TiZrHfCoNiCu; the inset shows the as-cast TiZrHfCoNiCu microstructure; (**b**) red line—Ni_67_Al_33_ and blue line—Co_22.33_Ni_22.33_Cu_22.33_Al_11_Ga_11_In_11_; the inset shows the as-cast Co_22.33_Ni_22.33_Cu_22.33_Al_11_Ga_11_In_11_.

Figure 1a shows a comparison of DTA crystallization curves for TiNi binary and Ti_16.67_Zr_16.67_Hf_16.67_Co_16.67_Ni_16.67_Cu_16.67_ multicomponent equiatomic compounds. It can be seen that binary equiatomic TiNi exhibit a single peak of crystallization with a width of 65 K that starts at 1587 K. The crystallization of the equiatomic Ti_16.67_Zr_16.67_Hf_16.67_Co_16.67_Ni_16.67_Cu_16.67_ compound starts at a lower temperature of 1530 K and the major DTA peak associated with it has a width of about 120 K. Further cooling reveals a small peak around 1200 K.

In Figure 1b, it can be seen that crystallization of the non-stoichiometric Ni_67_Al_33_ compound shows a two-stage behavior. The primary crystals of the Ni_67_Al_33_ compound begin to precipitate from the liquid phase at a temperature of 1733 K. The appearing crystals are still at equilibria with the liquid until 1687 K. As temperature decreases, the compositions of the crystals and the liquid change and the crystals become enriched in Ni. Finally, at 1687 K all of the liquid has solidified and there are areas in the as-cast microstructure that are different in composition but all of them belong to the same compound. In the case of crystallization of the Co_22.33_Ni_22.33_Cu_22.33_Al_11_Ga_11_In_11_ composition, the major crystallization event starts at 1500 K and, the crystallization process appears qualitatively similar to the binary Ni_67_Al_33_ compound but the crystallization peak width of 174 K is more than twice as wide as in the case of Ni_67_Al_33_ (75 K). Further cooling results in a two-stage event. The first stage starts at 1010 K, while the second one begins around 970 K (Figure 1b).

It has to be noted that crystallization of the equiatomic Ti_16.67_Zr_16.67_Hf_16.67_Co_16.67_Ni_16.67_Cu_16.67_ intermetallic compound takes place with the formation of stable (not undergoing a martensitic transformation) distorted B2 structure based on the X-ray data in [14,16], which show broad reflections. Some of that broadening mostly appears because of the strong dendritic liquation shown in the inset of Figure 1a. Against the background of the dendrites and interdendritic regions small black spots with the composition of (TiZrHf)_2_(CoNiCu) can be observed in the inset of Figure 1a.

In the case of CoNiCuAlGaIn, the equiatomic split was carried out in the frame of (CoNiCu) and (AlGaIn) groups of atoms (Co_22.33_Ni_22.33_Cu_22.33_Al_11_Ga_11_In_11_). Crystallization of this composition resulted in the formation of a (Co_31.7_Ni_26.8_Cu_8.5_)**_67_**(Al_22.1_Ga_10.3_In_0.6_)**_33_** phase surrounded preferentially by the (Co_6.3_Ni_13.5_Cu_48.9_)**_68.7_**(Al_1.5_Ga_4.5_In_25.3_)**_31.3_** phase of Cu_9_In_4_ type. These findings are in agreement with the results [17]. In addition, small amounts of the (Co_29.7_Ni_29.9_Cu_14.9_)**_73.6_**(Al_9.6_Ga_14.7_In_1.2_)**_26.4_** phase were observed, which could be distinguished from the previous one by SEM X-ray microanalysis.

It is well known that binary TiNi and NiAl intermetallic compounds of the considered stoichiometries exhibit appearance of martensitic microstructures upon cooling to room temperature. However, the equiatomic Ti_16.67_Zr_16.67_Hf_16.67_Co_16.67_Ni_16.67_Cu_16.67_ intermetallic compound [14] and the quasi-equiatomic (Co_22.33_Ni_22.33_Cu_22.33_Al_11_Ga_11_In_11_) (inset in Figure 1b) did not show any sign of martensite. In the case of the TiZrHfCoNiCu system, a variation of the Co, Ni, and Cu content was needed to introduce a martensitic transformation associated with shape memory behavior [15]. In particular, the composition Ti_16.67_Zr_16.67_Hf_16.67_Co_10_Ni_25_Cu_15_ has shown a martensitic transformation and shape memory at elevated temperatures. This composition was chosen in the present study for further detailed microstructure examination because of the possibility to observe martensite crystal formation against the dendritic liquation background. Similarly, in Reference [17] the general composition of Co_31.22_Ni_29.26_Cu_11.95_Al_16.64_Ga_10.39_In_0.55_ has demonstrated high temperature martensitic transformation and, as a result, a certain amount of martensite crystals have been formed against the background of a strong dendritic liquation.

**Figure 2:**
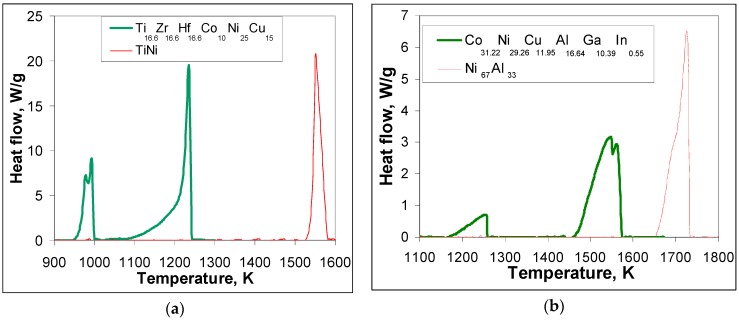
Heat flow upon crystallization from the liquid and further cooling (40 K/min cooling rate) of binary and multicomponent compounds: (**a**) red line—equiatomic TiNi and blue one—non-equiatomic Ti_16.67_Zr_16.67_Hf_16.67_Co_10_Ni_25_Cu_15_; (**b**) red line—Ni_67_Al_33_ and blue line—Co_31.22_Ni_29.26_Cu_11.95_Al_16.64_Ga_10.39_In_0.55_.

Figure 2 shows heat releases upon cooling from the liquid state followed by crystallization for Ti_16.67_Zr_16.67_Hf_16.67_Co_10_Ni_25_Cu_15_ (Figure 2a) and Co_31.22_Ni_29.26_Cu_11.95_Al_16.64_Ga_10.39_In_0.55_ multicomponent intermetallic compounds (Figure 2b) as compared to equiatomic TiNi (Figure 2a) and non-stoichiometric Ni_67_Al_33_ (Figure 2b), respectively.

It can be seen (Figure 2a) that the crystallization temperature for the not quite equiatomic composition Ti_16.67_Zr_16.67_Hf_16.67_Co_10_Ni_25_Cu_15_ is lower (1250 K) compared with the equiatomic one (1587 K, Figure 1a). In other words, the decrease in crystallization temperature as compared to binary TiNi is much higher for Ti_16.67_Zr_16.67_Hf_16.67_Co_10_Ni_25_Cu_15_ (337 K) than for equiatomic Ti_16.67_Zr_16.67_Hf_16.67_Co_16.67_Ni_16.67_Cu_16.67_ (only 57 K). The width of the crystallization peak is also 2.5 times larger for the non-equiatomic compound (164 K, Figure 2a) than for TiNi (65 K Figure 1a and Figure 2a), while in the case of the equiatomic one it is just 1.8 times as wide. Further cooling in the already solid state below 1090 K results in two overlapping peaks starting at 1006 K and 987 K, which have a width together of about 50 K.

In the case of non-stoichiometric Co_31.22_Ni_29.26_Cu_11.95_Al_16.64_Ga_10.39_In_0.55_ deviation from the initial composition resulted not only in the appearance of a martensitic transformation [17] but also in an increase in crystallization temperature from 1500 K in Co_22.33_Ni_22.33_Cu_22.33_Al_11_Ga_11_In_11_ (Figure 1b) to 1580 K (Figure 2b). In Figure 2b, the width of the peak is more narrow (115 K) than for Co_22.33_Ni_22.33_Cu_22.33_Al_11_Ga_11_In_11_ (174 K, Figure 1b). Further cooling from 1465 K revealed one prolonged peak. It starts at 1263 K (width about 90 K).

**Figure 3:**
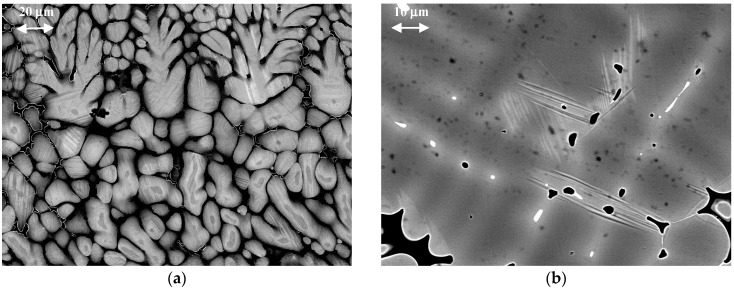
SEM as-cast microstructures observed using back-scattered electrons contrast: (**a**) Ti_16.67_Zr_16.67_Hf_16.67_Co_10_Ni_25_Cu_15_ and (**b**) Co_31.22_Ni_29.26_Cu_11.95_Al_16.64_Ga_10.39_In_0.55_.

Figure 3a,b show room temperature as-cast microstructures for Ti_16.67_Zr_16.67_Hf_16.67_Co_10_Ni_25_Cu_15_ and Co_31.22_Ni_29.26_Cu_11.95_Al_16.64_Ga_10.39_In_0.55_ compounds, respectively.

It is obvious that in both cases shown in Figure 3, the formation of the martensite phase takes place against the background of dendritic liquation. The interdendritic regions are decorated by pores and the formation of the phases occurs in an inhomogeneous matrix. Clearly, improved processing could address these issues and help to fully exploit the potential of these alloys.

In the case of Ti_16.67_Zr_16.67_Hf_16.67_Co_10_Ni_25_Cu_15_, the whole area of dendrites seems to be filled by martensite crystals of mostly plate like morphology (Figure 3a). Some of the interdendritic regions can be distinguished from the dendrites by the rather sharp contrast between them.

Figure 3b represents a different microstructure that forms in Co_31.22_Ni_29.26_Cu_11.95_Al_16.64_Ga_10.39_In_0.55_. First, the contrast between dendrites and interdendritic regions is much less, as compared to Ti_16.67_Zr_16.67_Hf_16.67_Co_10_Ni_25_Cu_15_ (Figure 3a), and, secondly, martensitic crystals have formed only in interdendritic regions seemingly being stopped from entering the dendrites. In addition, there is a more subtle difference between the microstructures in Figure 3a,b. The dendrites in Figure 3a appear brighter than the interdendritic regions, while in Figure 3b the situation is vice versa. Moreover, the second phase in the interdendritic region in Figure 3a appears black, while in Figure 3b it is white.

**Figure 4:**
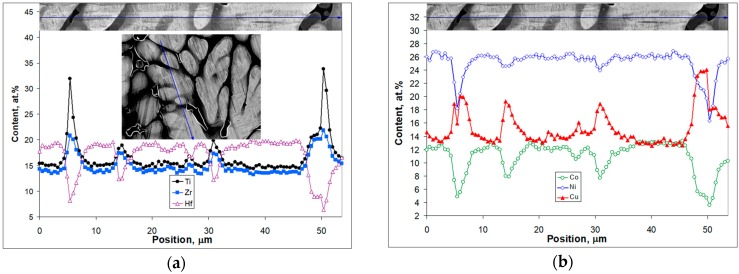
SEM images using back-scattered electron contrast and corresponding EDX line scan analysis for as-cast Ti_16.67_Zr_16.67_Hf_16.67_Co_10_Ni_25_Cu_15_: (**a**) Ti (closed circles), Zr (closed rectangles), Hf (open triangles) concentration profiles along the line shown in the upper inset; the central inset displays an overview of the microstructure along with line that is also shown in the upper inset; (**b**) Co (opened circles), Ni (opened rectangles), Hf (closed triangles) concentration profiles along the line shown in the upper inset.

The inset in the center of Figure 4a demonstrates the as-cast Ti_16.67_Zr_16.67_Hf_16.67_Co_10_Ni_25_Cu_15_ microstructure. The arrowed line appearing in both insets marks the same position, which was used to record EDX line scan data. The upper part of Figure 4a represents a higher magnification detail of the microstructure alongside the arrowed line that corresponds to the concentration profiles of Ti, Zr and Hf obtained from the EDX analysis. Figure 4b is structured similarly but represents the data for the Co, Ni and Cu concentration profiles. The line scan goes across two black precipitates, which are several micrometer thick and follows a martensite crystal that starts from one precipitate and stops at the other. Black in the back-scattered electron image corresponds to the area with lowest average atomic number (so-called Z contrast). From the scan data it was determined that the composition of the black appearing phase is (Ti_32_Zr_21.5_Hf_8.5_)**_62_**(Co_4_Ni_17_Cu_17_)**_38_**, which is in accordance with the findings shown in Figure 1a, where the phase of the Ti_2_Ni type was also found. On the right hand side of the scan (positioned at 45–49 μm in Figure 1) there is another area that is brighter and possesses a composition of (Ti_21_Zr_20_Hf_9_)_50_(Co_5_Ni_21_Cu_24_)_50_. The latter is the one that seems to be belonging to the B2 phase as no martensite crystals are seen in it. Yet, both compositions are not the major ones in the scan under consideration. Brighter patches about 10 μm long (upper inset in Figure 4) belong to dendrites with the composition (Ti_14.7_Zr_13.6_Hf_19.5_)_47.8_(Co_13_Ni_26.2_Cu_13_)_52.2_ and are filled by martensitic crystals (Figure 4a, central inset). The areas in between them that are few micrometer thick have the composition (Ti_19_Zr_17.4_Hf_12.4_)_48.8_(Co_7.9_Ni_24.4_Cu_18.9_)_52.2_, which is just in between the composition of the dendrites and dark appearing area ((Ti_21_Zr_20_Hf_9_)_50_(Co_5_Ni_21_Cu_24_)_50_).

**Figure 5:**
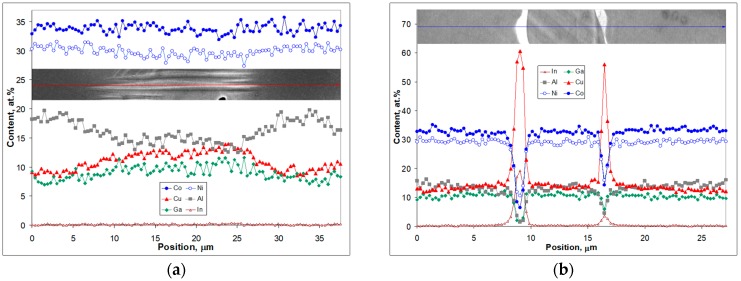
SEM images along with EDX line scan analysis for as-cast Co_31.22_Ni_29.26_Cu_11.95_Al_16.64_Ga_10.39_In_0.55_: (**a**) concentration profiles for all elements along the arrowed line recorded along and beyond the individual martensite crystal; (**b**) concentration profiles for all elements along the arrowed line recorded along the center of the interdendritic region.

Figure 5 shows SEM results obtained in a similar manner for the as-cast Co_31.22_Ni_29.26_Cu_11.95_Al_16.64_Ga_10.39_In_0.55_ microstructure.

It can be seen that the scan line in Figure 5a starts in a dark appearing region belonging to a dendrite and enters a brighter interdendritic region filled with martensite crystals. It passes through an individual 20-μm long martensite crystal, which stays within the interdendritic region. At the position where the scan line enters another dark appearing dendrite, the martensite crystal seems to stop from entering it. Other martensite crystals nearby display similar behavior (see also Figure 3b). Figure 5b shows a scan that goes through an interdendritic region. It crosses two bright appearing phases, which according to the data shown in Figure 5b have a composition of Co_6.4_Ni_10.6_Cu_60.7_Al_1.4_Ga_1.7_In_19.2_. This is close to the one of the Cu_9_In_4_ type found in [17]. The average composition of the dendrites shown in Figure 5a is (Co_33.9_Ni_30.5_Cu_9.4_)_73.8_(Al_18.2_Ga_7.9_In_0.1_)_26.2_. It changes smoothly in Figure 5a, as the scan is moving into the interdendritic area and undergoes a clear decrease in Al content, slight decrease in Co and Ni and a definite increase in Cu and Ga. The average composition of the interdendritic region extracted from the data in Figure 5b is (Co_32.7_Ni_29.4_Cu_13.3_)_75.4_(Al_13.8_Ga_10.6_In_0.2_)_24.6_.

Apparently, in both cases (TiZrHfCoNiCu and CoNiCuAlGaIn) the martensite crystals appear only in specific regions, which have a composition that best suit their respective stability as compared to the austenite B2 phase. The key difference is that for TiZrHfCoNiCu the martensite forms in dendrites, while for CoNiCuAlGaIn it forms in the interdendritic regions. In the following, the results concerning the crystallization process, formation of chemical inhomogeneities, and further formation of martensite crystals against the background of the inhomogeneities observed for the multicomponent TiZrHfCoNiCu and CoNiCuAlGaIn HESMA will be addressed in more detail.

## 4. Discussion

It is clear that the crystallization of multicomponent intermetallic compounds of the TiZrHfCoNiCu and CoNiCuAlGaIn type is quite different from the crystallization of binary TiNi and NiAl (Figure 1 and Figure 2). The crystallization temperatures are lower for the multicomponent compounds and the temperature intervals of crystallization are much wider. In the case of equiatomic TiZrHfCoNiCu, nine possible binary equiatomic compositions (corresponding to the compounds TiCo, ZrCo, HfCo, TiNi, ZrNi, HfNi, TiCu, ZrCu, and HfCu) crystallize together. As a result, the decrease in temperature of their joint solidification and the widening of their mutual crystallization temperature interval are expected. The solidus temperatures for all mentioned binaries were extracted from binary phase diagrams [21] and are summarized in Table 1 along with estimated decreases in crystallization temperature.

It can be seen that HfCo demonstrates the highest solidus temperature. The estimation of the decrease in solidus temperature for each of the nine AB compositions can be expressed as Δ*T*_sol_ = (*T*_HfCo-sol_ − *T*_AB-sol_)/9 and the solidification temperature of the multicomponent composition is obtained by the subtraction of the sum of the individual decreases for each AB composition from the solidus temperature of HfCo (*T*_cryst_ = *T*_HfCo-sol_ − (ΣΔ*T*_sol_). For the exact equiatomic composition Ti_16.67_Zr_16.67_Hf_16.67_Co_16.67_Ni_16.67_Cu_16.67_ it is 1535 K (Table 1), which is almost the same as the experimental value of 1530 K (Figure 1a). In the case of the Ti_16.67_Zr_16.67_Hf_16.67_Co_10_Ni_25_Cu_15,_ the solidus temperatures were taken for each binary composition from phase diagrams in [21] taking into account the deficiency in Co and the excess in Ni and Cu as compared with the equiatomic Ti_16.67_Zr_16.67_Hf_16.67_Co_16.67_Ni_16.67_Cu_16.67_ composition. The calculated value for the crystallization temperature of Ti_16.67_Zr_16.67_Hf_16.67_Co_10_Ni_25_Cu_15_ compound is 1264 K (Table 1), which is again quite close to the experimental value of 1250 K (Figure 2a).

The same consideration was applied for the non-stoichiometric CoNiCuAlGaIn multicomponent intermetallic compounds. In this case, amongst the nine possible binary equiatomic compositions (corresponding to compounds CoAl, CoGa, CoIn, NiAl, NiGa, NiIn, CuAl, CuGa, and CuIn) the highest solidus temperature is demonstrated by the equiatomic CoAl (*T*_CoAl-sol_ = 1913 K [21]), which is, by the way, the same as the one for HfCo. The calculated crystallization temperature for Co_22.33_Ni_22.33_Cu_22.33_Al_11_Ga_11_In_11_ is 1485 K (Table 1) and the experimental value is 1500 K (Figure 1b), which is not too far off. The change in general stoichiometry from 67:33 in (Co_22.33_Ni_22.33_Cu_22.33_)**_67_**(Al_11_Ga_11_In_11_)**_33_** to 73:27 in (Co_31.22_Ni_29.26_Cu_11.95_)**_73_**(Al_16.64_Ga_10.39_In_0.55_)**_27_** is also accompanied by a drastic decrease in In content to the point where the binary composition can be taken out from the consideration (Table 1). The calculated value for the crystallization temperature of (Co_31.22_Ni_29.26_Cu_11.95_)**_73_**(Al_16.64_Ga_10.39_In_0.55_)**_27_** is 1593 K (Table 1), which is close to the experimental value of 1580 K (Figure 2b).

In both cases described above that demonstrated such a good fit of experimental and calculated values of crystallization temperatures, the HfCo and CoAl intermetallic compounds (being the most refractory ones) were considered as the major constituents with respect to crystallization, while other binaries were assumed to just follow during the crystallization process. It also means that the final microstructures should have dendrites with excess of HfCo or CoAl in their composition, as compared to the interdendritic regions, which should be enriched in lower melting point constituents.

It should be noted that the DTA results (Figure 1 and Figure 2) show additional exothermal peaks upon further cooling following the crystallization process. These are indications of certain phase transformations that add up to the formation of the final microstructures. Generally, this means that the stabilizing effect of the higher entropy of mixing decreases with decreasing temperature and at certain point precipitation of second phases follows. To consider this situation more properly, one needs to have an idea about the contribution of enthalpy and entropy of mixing. Both values for all multicomponent intermetallics considered have been summarized in Table 2.

In the case of equiatomic TiZrHfCoNiCu shown in Figure 1a, the weak exothermic peak around 1200 K corresponds to the precipitation of the (TiZrHf)_2_(CoNiCu) phase, which takes place in the interdendritic region. The volume fraction of these precipitates is rather small. Still, they appear in interdendritic regions despite the high values for the enthalpy of mixing, Δ*H*_mix_ = −29.9 kJ/mol, and the mixing entropy, Δ*S*_mix_ = 14.897 Jmol^−1^K^−1^ (Table 2).

The non-stoichiometric (Co_22.33_Ni_22.33_Cu_22.33_)(Al_11_Ga_11_In_11_) has a rather low enthalpy of mixing value, Δ*H*_mix_ = −2.2 kJ/mol, while the entropy of mixing is considerably high, Δ*S*_mix_ = 14.407 Jmol^−1^K^−1^ (Table 2). Therefore, this multicomponent intermetallic compound crystallizes supposedly into a dendritic microstructure similar to the one shown in Figure 1a and remains single phase upon cooling down to 1000 K temperature where it decomposes into two phases (Figure 1b), which are different from the high temperature dendritic one. Cooling by 300 K, to 1000 K, was enough to diminish the entropy contribution to the point where the single phase lost its stability and decomposed. The final microstructure in this case is then a two phase mixture. It seems like the dendrites being depleted in Cu and In might even release some of them into the interdendritic regions, which were already enriched by these. This then grew into separate phases because the interdendritic regions in their turn are depleted in Co, Ni, Al, and Ga and their additional diffusion takes place upon precipitation. As a result, two exothermal peaks can be observed corresponding to the formation of two new phases (Figure 1b). The black appearing grains of the (Co_31.7_Ni_26.8_Cu_8.5_)**_67_**(Al_22.1_Ga_10.3_In_0.6_)**_33_** phase (60% of the volume fraction) are indeed depleted in Cu and In and are surrounded by a phase with the composition (Co_6.3_Ni_13.5_Cu_48.9_)_68.7_(Al_1.5_Ga_4.5_In_25.3_)_31.3_, which is depleted in Co, Ni, Al, and Ga.

**Figure 6:**
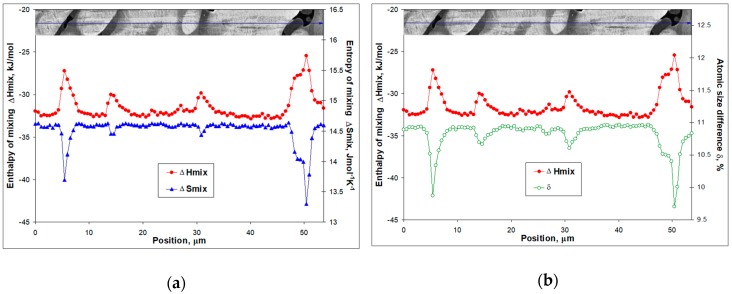
Changes along the EDX line scan shown in Figure 4 within the as-cast Ti_16.67_Zr_16.67_Hf_16.67_Co_10_Ni_25_Cu_15_ for: (**a**) enthalpy (closed circles) and entropy (closed triangles) of mixing calculated as described for Table 2 according to [22,23]; (**b**) enthalpy of mixing (closed circles) and atomic size difference (open circles) calculated according to [23].

The Ti_16.67_Zr_16.67_Hf_16.67_Co_10_Ni_25_Cu_15_ compound also underwent decomposition upon cooling down by about 100 K below crystallization (Figure 2a). In this case, the enthalpy of mixing is even higher (Δ*H*_mix_ = −31.9 kJ/mol, Table 2), while the entropy of mixing is only slightly lower Δ*S*_mix_ = 14.61 Jmol^−1^K^−1^ (Table 2) as compared to the equiatomic composition. Two peaks indicate the formation of (Ti_32_Zr_21.5_Hf_8.5_)**_62_**(Co_4_Ni_17_Cu_17_)**_38_**, which is similar to (TiZrHf)_2_(CoNiCu) case observed in microstructures of the equiatomic compound, and to the formation of (Ti_21_Zr_20_Hf_9_)**_50_**(Co_5_Ni_21_Cu_24_)**_50_** phase (Figure 4). To explain this precipitation process from a seemingly stable (at least according to the high values of enthalpy and entropy of mixing) high temperature phase and formation of martensite crystals within dendrites upon further cooling (cf. Figure 3a and Figure 4), one needs to have a closer look at the values in Table 2. The enthalpy of mixing and the entropy of mixing to a certain extent represent an average measure of the interatomic interaction in the multicomponent intermetallic compound under consideration. In fact, it appears that the interatomic interaction in the highly distorted lattice undergoes a continuous changes upon cooling and represents a key to understanding the competing phase transformations (diffusion and diffusionless). A detailed analysis of these interactions is under way. In the present study, some additional information is extracted using parameters that are average in nature to characterize the collective behavior in the multicomponent alloys. The results for the as-cast Ti_16.67_Zr_16.67_Hf_16.67_Co_10_Ni_25_Cu_15_ are shown in Figure 6.

The dependencies in Figure 6 show a clear anti correlation. The correlation coefficient for the enthalpy and entropy of mixing pair is −0.87 (Figure 6a), while the enthalpy of mixing and the atomic size difference show an even-better anti correlation (correlation coefficient is −0.95; Figure 6b). First of all, this means that lattice distortions are followed more accurately by the atomic size difference and the enthalpy of mixing no matter how average these values are. The entropy of mixing calculated here according to the Boltzmann equation reflects chemical changes only. On the other hand, it can be seen that the dendrite region shows the highest entropy of mixing and atomic size difference. This is the region where the martensite crystals actually appeared. The interdendritic regions are unreachable for martensite although there is no clear boundary between the interdendritic regions and the dendrites. The stabilization of these regions with respect to the martensitic transformation is accompanied by lower negative values of the enthalpy of mixing, lower entropy of mixing and change in atomic size difference. In other words, the martensite crystals are not able to penetrate the less distorted interdendritic regions that are also less stable. Here the loss in stability can be related to diffusional phase transformations, which have been confirmed by the precipitation of (TiZrHf)_2_(CoNiCu) in these regions as detected by DTA (Figure 1a and Figure 2a) and observed by SEM (Figure 3a, Figure 4 and Figure 6).

**Figure 7:**
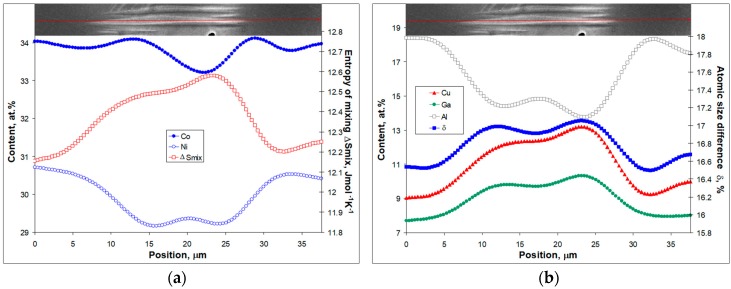
Variation of key parameters along the EDX line scan shown in Figure 5a within the as-cast Co_31.22_Ni_29.26_Cu_11.95_Al_16.64_Ga_10.39_In_0.55_: (**a**) smoothened concentration profiles of Co (closed circles), Ni (open circles) and entropy of mixing calculated as described for Table 2 according to [23] (open boxes); (**b**) smoothened Cu (closed triangles), Ga (closed circles), Al (open boxes) concentration profiles, and atomic size difference (closed boxes) calculated according to [23].

Next it is considered how the same line of thinking can be applied to the case of the as-cast Co_31.22_Ni_29.26_Cu_11.95_Al_16.64_Ga_10.39_In_0.55_ intermetallic compound. The description given previously for the results in Figure 5 will be expanded here using the data given in Figure 5a. To analyze the concentration profiles, they were first smoothened as described in Section 2. Smoothened concentration variations for Co, Ni, Cu, Al, and Ga along a 38 μm scan are plotted in Figure 7. In addition, entropy of mixing and atomic size difference are included in Figure 7 as well. For the sake of clarity, the enthalpy of mixing dependence and the In concentration profile are not shown. The enthalpy of mixing correlates extremely well with the Cu concentration profile (correlation coefficient is 0.99) as well as with the entropy of mixing (correlation coefficient is 0.99).

Figure 7 shows all the variation in the key parameters with the as-cast microstructure and near perfect correlations of the Ga concentration profile with atomic size difference (correlation coefficient is 0.97) and Ni with Al (correlation coefficient is 0.95). In other words, dendrite regions (dark appearing ones on the right and left hand sides of the interdendritic region shown in Figure 5a and Figure 7) are enriched in CoAl and NiAl as those started to crystallize from the most refractory compounds (see Table 1). As the scan is followed into the interdendritic region, a depletion in Ni (Figure 7a) and Al (Figure 7b) becomes apparent. It should be noted that martensite crystal displays similar trends at this position. At the same time, lower melting point constituents like Cu, Ga, and In are enriched in interdendritic regions instead of Ni, Al, and Co. Still, the Co concentration profile does not correlate well with any of the other dependencies seen in Figure 7. It goes through a maximum, which is followed by a minimum. This concentration changes only by about one at %, which can be the reason why martensite crystal did not stop when coming through the Co concentration wave shown in Figure 7a. Other elements (except In) undergo stronger changes coming from the dendrite into the interdendritic region.

Comparing this case with TiZrHfCoNiCu (Figure 6), it should be noted that martensite crystals appear similarly in the regions that are more distorted (atomic size difference increases—Figure 7b) and the enthalpy of mixing has a lower negative value. However, one more important difference has to be noticed. The region where the martensitic crystals appears is not a dendrite but an interdendritic one. Moreover, the entropy of mixing (Figure 7a) undergoes an increase in this region as well as the atomic size difference (Figure 7b). Once again, similarly to TiZrHfCoNiCu, the martensite crystals propagate through highly distorted regions but are not able to penetrate less distorted regions of the CoNiCuAlGaIn intermetallic compound. The only difference is that in the former case martensite forms in dendrites, while in the latter—in interdendritic regions. These findings are in agreement with the results reported in Reference [16], where it was concluded that the distorted crystal structure of TiZrHfCoNiCu intermetallic compounds is even better prepared for martensitic transformation than binary TiNi or ZrCu.

## 5. Summary and Outlook

The high entropy shape memory alloys (HESMA) studied in the present paper feature a martensitic transformation and shape memory behavior in the as-cast state [14,15,16,17]. The present study has shown that this state is characterized by strong dendritic liquation that develops into similar multi-phase microstructures. Yet two different crystallization scenarios evolve that result in the formation of martensite in dendrites or interdendritic regions. Control of the crystallization parameters might allow one to refine the final microstructures to obtain cast and ready to use HESMA without subsequent solutionizing. This might become extremely useful, especially in the case of 3D printing using HESMA. It should be noted that following the first HESMA breakthrough described in References [14,15,16,17], in these publications where solutionized composition Ti_16.67_Zr_16.67_Hf_16.67_Co_10_Ni_25_Cu_15_ was studied [15,16]. Yet, quite significant remnants of Ti_2_Ni like precipitates were observed in the microstructure after solution treatment [24,25]. The present findings clearly show the inevitability of such precipitations as interdendritic regions are provide template-like sites for those precipitates. Obviously, such precipitations will affect the functional properties as it was shown already for the TiNi binary system. The authors of References [24,25] have shown that starting at 200 MPa applied external stress, the shape memory effects start to degrade upon fatigue of solutionized Ti_16.67_Zr_16.67_Hf_16.67_Co_10_Ni_25_Cu_15_. Amongst other possible reasons for such behavior might be the presence of precipitates. It is clear that simple solution treatments cannot fully avoid their formation. Realization of a homogeneous microstructure suitable for fully reversible martensitic transformation associated with a stable-shape memory behavior will be challenging. In fact, a single-phase material might not be the best solution in this case.

## Figures and Tables

**Table 1:** Solidus temperatures (*T*_sol_) for binary compounds with different stoichiometries taken from binary phase diagrams [21]; decrease in solidus temperature estimation for nine AB compositions comparing with the composition with the highest solidus (HfCo for systems ^1^ and ^2^ Δ*T*_sol_ = (*T*_HfCo-sol_ − *T*_AB-sol_)/9; Co_67_Al for ^3^ Δ*T*_sol_ = (*T*_Co67Al-sol_ − *T*_A67B-sol_)/9 and Co_73_Al for ^4^ Δ*T*_sol_ = (*T*_Co73Al-sol_ − *T*_A73B-sol_)/6) within the multicomponent high entropy compound; crystallization temperatures for multicomponent high entropy compounds calculated as *T*_cryst_^1,2^ = *T*_HfCo-sol_ − (ΣΔ*T*_sol_) and *T*_cryst_^3,4^ = *T*_CoAl-sol_ − (ΣΔ*T*_sol_).

	**TiCo**	**ZrCo**	**HfCo**	**TiNi**	**ZrNi**	**HfNi**	**TiCu**	**ZrCu**	**HfCu**	***T*_cryst_^HEA^, K**
^1^*T*_sol_, K	1598	1673	1913	1586	1533	1803	1251	1208	1253	1535
^1^Δ*T*_sol,_ K	35	26.66	0	36.33	42.22	12.22	73.55	78.33	73.33	
^2^*T*_sol,_ K	1508	1585	1823	1239	1283	1466	1206	1163	1243	1264
^2^Δ*T*_sol,_ K	52.5	39.66	0	97.33	90	59.5	102.83	110	96.66	
	**Co_67_Al**	**Co_67_Ga**	**Co_67_In**	**Ni_67_Al**	**Ni_67_Ga**	**Ni_67_In**	**Cu_67_Al**	**Cu_67_Ga**	**Cu_67_In**	***T*_cryst_^HEA^, K**
^3^*T*_sol,_ K	1849	1483	1559	1778	1483	1223	1311	1098	938	1485
^3^Δ*T*_sol,_ K	0	40.666	32.22	7.888	40.666	69.55	59.77	83.44	101.2	
	**Co_73_Al**	**Co_73_Ga**	**Co_73_In**	**Ni_73_Al**	**Ni_73_Ga**	**Ni_73_In**	**Cu_73_Al**	**Cu_73_Ga**	**Cu_73_In**	***T*_cryst_^HEA^, K**
^4^*T*_sol,_ K	1773	1526	-	1668	1480	-	1318	953	-	1593
^4^Δ*T*_sol,_ K	0	41.166	-	17.5	48.833	-	75.833	136.66	-	

^1^ Ti_16.67_Zr_16.67_Hf_16.67_Co_16.67_Ni_16.67_Cu_16.67_, ^2^ Ti_16.67_Zr_16.67_Hf_16.67_Co_10_Ni_25_Cu_15_, ^3^ Co_22.33_Ni_22.33_Cu_22.33_Al_11_Ga_11_In_11_, ^4^ Co_31.22_Ni_29.26_Cu_11.95_Al_16.64_Ga_10.39_In_0.55_. *T*_CoAl-sol_ = *T*_HfCo-sol_ = 1913 K [21].

**Table 2:** Enthalpies of mixing (Δ*H*_mix_) calculated in [22] by Miedema’s model for atomic pairs and calculated in the present study for TiZrHfCoNiCu (^1^ and ^2^ compositions) and CoNiCuAlGaIn (^3^ and ^4^ compositions) according to [23]; corresponding entropies of mixing a provided in the footnote.

	**TiCo**	**ZrCo**	**HfCo**	**TiNi**	**ZrNi**	**HfNi**	**TiCu**	**ZrCu**	**HfCu**	**NiCu**	**CoCu**				**HEA**
Δ*H*_mix,_ kJ/mol	−28	−41	−35	−35	−49	−42	−9	−23	−17	4	6				^1^ −29.9
															^2^ −31.9
	**CoAl**	**CoGa**	**CoIn**	**NiAl**	**NiGa**	**NiIn**	**CuAl**	**CuGa**	**CuIn**	**NiCu**	**CoCu**	**AlGa**	**AlIn**	**GaIn**	**HEA**
Δ*H*_mix,_ kJ/mol	−19	−11	7	−22	1	2	−1	−15	10	4	6	1	7	3	^3^ −2.2
															^4^ −8.8

^1^ Ti_16.67_Zr_16.67_Hf_16.67_Co_16.67_Ni_16.67_Cu_16.67_ (Δ*S*_mix_ = 14.897 Jmol^−1^K^−1^ [14]), ^2^ Ti_16.67_Zr_16.67_Hf_16.67_Co_10_Ni_25_Cu_15_ (Δ*S*_mix_ = 14.61 Jmol^−1^K^−1^), ^3^ Co_22.33_Ni_22.33_Cu_22.33_Al_11_Ga_11_In_11_ (Δ*S*_mix_ = 14.407 Jmol^−1^K^−1^), ^4^ Co_31.22_Ni_29.26_Cu_11.95_Al_16.64_Ga_10.39_In_0.55_ (Δ*S*_mix_ = 12.796 Jmol^−1^K^−1^). All mixing entropies were calculated according to the Boltzmann equation as in [23].
